# Susceptibility to ATP depletion of primary proximal tubular cell cultures derived from mice lacking either the α1 or the α2 isoform of the catalytic domain of AMPK

**DOI:** 10.1186/1471-2369-14-251

**Published:** 2013-11-14

**Authors:** Wilfred Lieberthal, Meiyi Tang, Leiqing Zhang, Benoit Viollet, Vimal Patel, Jerrold S Levine

**Affiliations:** 1 Department of Medicine, Stony Brook University Medical Center, Stony Brook, NY 11794, USA; 2 Northport Veterans Affairs Medical Center, Northport, NY 11768, USA; 3 Inserm, U1016, Institut Cochin, Paris, France; 4 CNRS, UMR 8104, Paris, France; 5 Université Paris Descartes, Sorbonne Paris Cité, Paris, France; 6 Department of Medicine, University of Illinois at Chicago, Chicago, IL 60612, USA; 7 Jesse Brown Veterans Affairs Medical Center, Chicago, IL 60612, USA

**Keywords:** AMPK, Viability, Survival, Apoptosis knockout mice, shRNA, ATP depletion, Metabolic stress, Antimycin

## Abstract

**Background:**

The purpose of this study was to determine whether AMPK influences the survival of primary cultures of mouse proximal tubular (MPT) cells subjected to metabolic stress. Previous studies, using an immortalized MPT cell line, suggest that AMPK is activated during metabolic stress, and ameliorates stress-induced apoptosis of these cells.

**Methods:**

Primary MPT cells were cultured from AMPK knockout (KO) mice lacking either the α1 or the α2 isoform of the catalytic domain of AMPK. MPT cells were subjected to ATP depletion using antimycin A.

**Results:**

Surprisingly, there was no difference in the amount of death induced by metabolic stress of MPT cells from either type of AMPK KO mice compared to its WT control. Moreover, inhibition of the activity of the α1 isoform in primary MPT cells from α2^-/-^ mice (pharmacologically, via compound C) or inhibition of the α2 isoform in primary MPT cells from α1^-/-^ mice (molecularly, via knockdown) both decreased cell viability equivalently in response to metabolic stress. The explanation for this unexpected result appears to be an adaptive increase in expression of the non-deleted α-isoform. As a consequence, total α-domain expression (i.e. α1 + α2), is comparable in kidney cortex and in cultured MPT cells derived from either type of KO mouse versus its WT control. Importantly, each α-isoform appears able to compensate fully for the absence of the other, with respect to both the phosphorylation of downstream targets of AMPK and the amelioration of stress-induced cell death.

**Conclusions:**

These findings not only confirm the importance of AMPK as a pro-survival kinase in MPT cells during metabolic stress, but also show, for the first time, that each of the two α-isoforms can substitute for the other in MPT cells from AMPK KO mice with regard to amelioration of stress-induced loss of cell viability.

## Background

AMP-activated protein kinase (AMPK) is a ubiquitously expressed and highly conserved serine/threonine kinase that is activated by any form of stress that reduces cell energy stores [1,2]. AMPK is a heterotrimeric protein composed of a catalytic α subunit and two regulatory (β and γ) subunits [1]. There are two isoforms of the catalytic (α1 and α2) and β (β1 and β2) subunits, and three isoforms of the γ (γ1, γ2, and γ3) subunit, all of which are encoded by different genes [1]. AMPK is activated by phosphorylation of a threonine residue (Thr^172^) situated within the activation loop of the α-subunit [3-5]. The best-defined upstream AMPK kinase is liver kinase B1 (LKB1). LKB1 is constitutively active, so that AMPK is continuously being phosphorylated [3-5]. When cell energy stores are replete, AMPK is maintained in an inactive state by the activity of phosphatases such as phosphatase-2C alpha [1,3,4]. Activation of AMPK occurs whenever cell stress leads to a decrease in cytosolic ATP levels and a concomitant increase in levels of adenosine diphosphate (ADP) and adenosine monophosphate (AMP) [3,4]. The regulatory γ-subunit of AMPK has binding sites for all three nucleotides [6,7]. Binding of ADP or AMP to the γ-subunit activates AMPK via two mechanisms, inhibition of the dephosphorylation of Thr^172^ and allosteric facilitation of phosphorylation of AMPK by LKB1 [3,4]. In contrast to the effects of ADP and AMP, binding of ATP to the γ subunit of AMPK promotes its deactivation [1,3,4]. The net result of these opposing interactions is that AMPK activity is inversely related to the ratio of the concentration of ATP to that of ADP and AMP [3,4,7].

Upon activation, AMPK phosphorylates and alters the activity of multiple downstream kinases and enzymes [8,9]. AMPK also induces changes in gene expression [10]. Together, these effects of AMPK lead to alterations in glucose, protein, and lipid metabolism that serve to conserve energy stores by promoting ATP production, inhibiting ATP consumption, and facilitating the cellular uptake of nutrients [1,2,8,9,11,12]. For example, through phosphorylation and inhibition of acetyl-CoA carboxylase 1 (ACC1) and ACC2, AMPK inhibits lipid synthesis and promotes fatty acid oxidation [1,13-15]. By inhibiting the activity of the mammalian target of rapamycin (mTOR), AMPK also inhibits protein synthesis [16,17]. Other AMPK-mediated effects include increased cellular uptake of glucose and fatty acids, and increased glycolysis [9].

Recently, using an immortalized mouse proximal tubular (MPT) cell line, we have shown that AMPK is activated by ATP depletion, and that, once activated, AMPK ameliorates apoptosis induced by this form of stress [18]. Here we extend these findings to primary cultures of MPT cells, derived from the kidneys of AMPK knockout (KO) mice with whole body deletions of either the α1 (α1^-/-^) or the α2 (α2^-/-^) isoforms of AMPK. We hypothesized that primary MPT cells, lacking one or other of the α-isoforms of AMPK, should be more susceptible to apoptosis induced by ATP depletion than primary MPT cells with both α-isoforms intact. Surprisingly, we found no difference in the severity of antimycin-induced cell death in MPT cells from α1^-/-^ or α2^-/-^ mice versus their WT controls. Moreover, we found that a reduction of AMPK activity (via either pharmacologic inhibition or knockdown of one of the α-isoforms) led to comparable increases of antimycin-induced apoptosis in primary MPT cells from KO versus WT mice.

In explaining this absence of a difference in stress-induced apoptosis for MPT cells from KO versus WT mice, we show that there is a compensatory up-regulation in expression of the intact α-isoform in both AMPK KO mice. This up-regulation compensates fully for the deleted α-isoform, such that the total amount of α-domain protein is comparable in primary MPT cells from the two KO mice versus their WT controls. These findings not only confirm the importance of AMPK activation as a pro-survival kinase during ATP depletion, but also indicate that each of the two α-isoforms can substitute for the other in ameliorating the consequences of metabolic stress.

## Methods

### Materials

All chemicals were purchased from Sigma (MO) unless otherwise stated. Soybean trypsin inhibitor was purchased from GIBCO (NY). Rabbit polyclonal antibody to total AMPK, rabbit polyclonal antibody to total alpha subunit of AMPK, and mouse monoclonal antibody to the phosphorylated alpha subunit of AMPK were purchased from Cell Signaling Technologies (MA). Rabbit polyclonal antibody to phosphorylated ACC1/2 was purchased from Upstate (NY). Rabbit polyclonal antibodies specific for the α1 and α2 isoforms of the catalytic domain of AMPK were purchased from Abcam (MA). Monoclonal antibody to β-actin was purchased from Chemicon (CA).

### Mice

The generation and phenotype of whole body α1^-/-^ and α2^-/-^ KO mice have been described previously [19,20]. α1^-/-^ mice and their WT controls were bred on a mixed background (C57BL6J/129Sv), whereas α2^-/-^ KO mice and their WT controls were bred on a C57BL6J background. Heterozygous α1^-/-^ and –α2^-/-^ KO mice, used only for breeding, were obtained from the European Mouse Mutant Archive and bred in the Department for Laboratory and Animal Research at Stony Brook Hospital. Only homozygous KO mice, male and female, were used for experimentation. All studies were approved by the IACUC at Stony Brook Hospital. Total body weights and kidney weights were comparable amongst KO and WT mice (data not shown).

### Primary culture of mouse proximal tubular (MPT) cells

Techniques for the primary culture of MPT cells are well established in our laboratory [21,22]. Briefly, kidneys were harvested from mice at 4–6 weeks of age. Cortical tissue was finely minced and incubated in Hanks’s solution containing collagenase and soybean trypsin inhibitor. After collagenase digestion, 10% horse serum was added, and the tissue fragments were gently centrifuged. After two washes with DMEM, tissue fragments were suspended in growth medium, a serum-free, defined medium consisting of DMEM/Ham’s F-12 media (1:1) containing 2 mM glutamine, 15 mM HEPES (pH 7.2), 5 μg/ml transferrin, 5 μg/ml insulin, 50 mM hydrocortisone, 500 U/ml penicillin, and 50 mg/ml streptomycin. The tissue fragments, suspended in growth medium, were then plated on plastic tissue cultures plates and incubated in a humidified air/CO_2_ incubator (5% v/v) at 37°C.

Fragments of tubules adhere to the culture dish, and MPT cells grow out of the tubules to form confluent monolayers over a 4–5 day period. MPT cells express megalin and other markers specific for proximal tubular cells. Five days after plating, the medium was changed to experimental medium, which consists of DMEM/Hams F12 containing HEPES (pH 7.2), 15 mM glutamine, 500 U/ml penicillin, and 50 mg/ml streptomycin. No insulin, hydrocortisone, or transferrin was added to this medium. Experiments were begun on the sixth day after plating.

### Metabolic stress (graded ATP depletion)

MPT cells were exposed to metabolic stress using antimycin A, a mitochondrial inhibitor. We examined the effect of antimycin A in the presence of three concentrations of dextrose, 10, 5, and 2.5 mM. The severity of metabolic stress increases progressively, (i.e. cell ATP levels fall progressively), as the dextrose concentration in the medium is reduced from 10 to 2.5 mM. We refer to this model of metabolic stress as “graded” ATP depletion. We have used graded ATP depletion as a model of metabolic stress in previous publications [22]. Control (unstressed) MPT cells were incubated in 5 mM dextrose in the absence of antimycin.

### Cell ATP levels

ATP levels were determined using previously described methods [22]. Briefly, cell ATP content was measured by luciferase assay in cell lysates and normalized to total cellular protein, as assessed by bicinchoninic acid (BCA) protein assay (Pierce, Rockford, NY). Control ATP levels, obtained in cells incubated in dextrose-containing medium (5 mM) in the absence of antimycin, were expressed as nanomoles of ATP per milligram of cell protein (nM ATP/mg protein). ATP levels obtained during metabolic stress were expressed as a percentage of ATP levels under control conditions.

### Immunoblotting

Immunoblotting was performed as previously described [23]. Briefly, cells were washed with ice-cold PBS, then lysed in ice-cold cell lysis buffer (20 mM Tris–HCl, pH 7.5, containing 130 mM NaCl, 1% v/v Triton X-100, 0.5% w/v deoxycholate, 0.1% w/v SDS, 1 mM DTT, 10 mM sodium pyrophosphate, 5 mM sodium fluoride, 1 mM phenylmethylsulfonyl fluoride, and 200 μM sodium orthovanadate). Lysates were centrifuged at 10,000 × *g* for 10 min at 4°C, and the supernatants were stored at -70°C. Protein samples, 20 μg per lane, as determined by BCA protein assay, were boiled in 6× reducing sample buffer, electrophoresed on SDS-polyacrylamide gels, and transferred to nitrocellulose membranes (BIO-RAD, Hercules, CA). Membranes were blocked with either 2.5% bovine serum albumin or 5% dry milk in TBS, before probing with primary antibody. After incubation with the appropriate secondary antibody, immunoreactive bands were visualized by the Western Lightning Chemiluminescence Reagent Plus (PerkinElmer, Boston, MA).

### Cell viability

Cell viability was determined using the LIVE/DEAD Assay Kit purchased from Molecular Probes™ and used according to the manufacturer’s instructions. In brief, MPT cells were stained with ethidium homodimer-1 (EthD-1) and calcein AM. Live cells are identified by their ability to convert calcein AM, a non-fluorescent cell-permeant agent to calcein, an intensely fluorescent dye (excitation/emission wavelengths, ~495 nm/~515 nm) that is retained within live cells. Dead cells are identified by nuclear staining for EthD-1, which only enters cells with damaged plasma membranes and, upon binding to nucleic acids, undergoes a 40-fold enhancement of fluorescence (excitation/emission wavelengths, ~495 nm/~635 nm), thereby producing a bright fluorescence in dead cells. Since both dyes are essentially non-fluorescent before interacting with cells, background fluorescence is inherently low. Live and dead cells were quantitated using flow cytometry (FACScan, BD Biosciences), and data were analyzed using CELLQuestPro Version 3.3 (BD Biosciences). Cells were first analyzed by forward versus side scatter, and gated to remove debris, cell fragments, and cell aggregates. The proportion of live cells in each sample was expressed as a percent of the total number of cells analyzed (10,000/sample).

### Statistics

All data are presented as mean ± standard error (SE). Student’s t-test was used for comparing cell ATP levels and densitometry of immunoblots. The Bonferroni correction was applied when multiple comparisons were made. The viability of MPT cells cultured from KO versus WT mice and subjected to metabolic stress was compared by ANOVA for repeated measures using STATA® Data Analysis and Statistical Software. All p values <0.05 were considered statistically significant.

## Results

### Effect of metabolic stress on the viability of MPT cells from α1^-/-^ and α2^-/-^ versus WT mice

**Figure 1:**
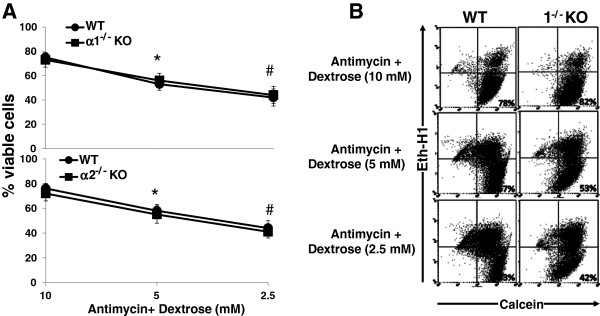
**Effect of metabolic stress on the viability of MPT cells from α1**^**-/- **^**and α2**^**-/- **^**KO mice. A**: MPT cells from α1^-/-^ mice (upper panel) or α2^-/-^ KO mice (lower panel), or their WT controls, were subjected to graded metabolic stress by incubation in the presence of antimycin (2 μM) and varying concentrations of dextrose. After 16–18 hrs, the percentage of viable cells remaining was determined by flow cytometry (n = 5 for both experiments). * p < 0.02, 5 mM dextrose vs. 10 mM dextrose; # p < 0.02, 2.5 mM dextrose vs. 5 mM dextrose. **B**: Representative flow cytometric analysis of MPT cells from α1^-/-^ mice (right panels) and their WT control (left panels) incubated in antimycin (2 μM) plus varying concentrations of dextrose. Cell viability was quantified by staining cells with calcein AM (x-axis) and ethidium homodimer-1 (Eth-H1) (y-axis). For each condition, 10,000 cells were analyzed, and the percentage of viable cells was calculated by dividing the number of calcein-positive and Eth-H1-negative cells by the total number of cells counted.

We determined the effect of graded ATP depletion, induced by exposing MPT cells to antimycin A and varying concentrations of dextrose, on cell viability, as assessed by flow cytometry. Cell viability was comparable in MPT cells from KO versus WT mice under unstressed control conditions (10 mM dextrose, no antimycin) (data not shown). In the presence of antimycin, the percentage of viable MPT cells from AMPK KO and WT mice decreased progressively as the concentration of dextrose was decreased (Figure 1). Nevertheless, at each dextrose concentration, the survival of MPT cells from α1^-/-^ or α2^-/-^ KO mice was no different than that of MPT cells from WT controls (Figure 1).

### Effect of metabolic stress on cytosolic ATP levels in MPT cells from α1^-/-^ and α2^-/-^ mice

**Figure 2:**
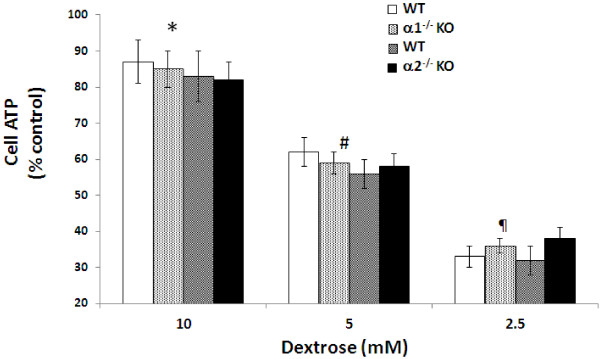
**Effect of metabolic stress on cytosolic ATP levels in MPT cells from α1**^**-/- **^**and α2**^**-/- **^**KO mice.** MPT cells from α1^-/-^ mice or α2^-/-^ KO mice, or their WT controls, were subjected to graded ATP depletion by incubation in the presence of antimycin (2 μM) and varying concentrations of dextrose for 4 hrs. Cell ATP levels were measured and normalized to cell protein content. All values are expressed as a percent of ATP levels in MPT cells incubated under control conditions (10 mM dextrose, in the absence of antimycin). * p < 0.02, antimycin plus 10 mM dextrose vs. control conditions; # p < 0.01, antimycin plus 5 mM dextrose vs. antimycin plus 10 mM dextrose; p < 0.01, antimycin plus 2.5 mM dextrose vs. antimycin plus 5 mM dextrose.

Cytosolic ATP levels in control unstressed MPT cells incubated in 10 mM dextrose without antimycin for 4 hrs were no different for KO versus WT controls (21 ±8 nM/mg protein versus 23 ± 6 nM/mg protein for α1^-/-^ mice; and 26 ± 9 nM/mg protein versus 21 ± 7 nM/mg protein for α2^-/-^ mice). Incubation of MPT cells in the presence of antimycin for 4 hrs led to a reduction in ATP levels. The severity of ATP depletion was inversely proportional to the concentration of dextrose in the medium (Figure 2). However, the severity of ATP depletion did not differ in MPT cells from KO versus WT mice at any dextrose concentration (Figure 2).

### Relative expression of the catalytic alpha domains of AMPK in whole kidney cortex and in cultured proximal tubular cells obtained from α1^-/-^ and α2^-/-^ KO and WT mice

**Figure 3:**
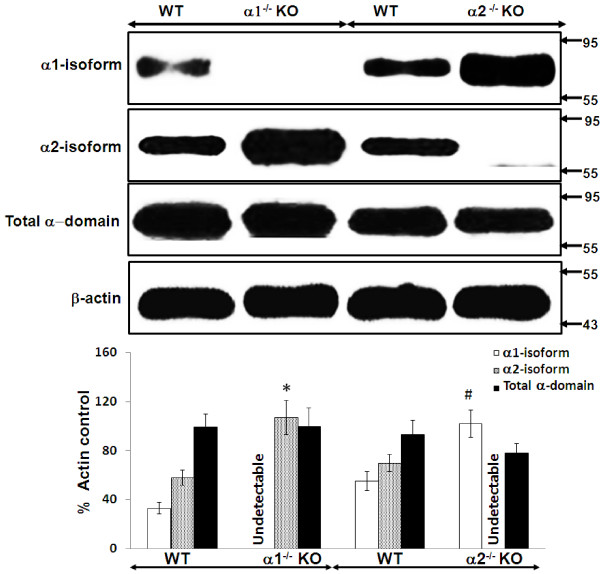
**Relative expression of the catalytic alpha (α) domain of AMPK in whole kidney cortex obtained from α1**^**-/- **^**and α**^**-/- **^**knockout (KO).** The relative expression of the α1 and α2 isoforms of AMPK, and of the total α(α1 + α2) domain, was evaluated in immunoblots of lysates of “snap frozen” samples of kidney cortex, obtained from α1^-/-^ or α2^-/-^ KO mice and their WT controls. Upper panel: Representative immunoblot. Molecular weight markers (in kDa) are shown on the right of each immunoblot. Lower panel: Densitometric quantitation of three immunoblots using β-actin as a loading control. * p < 0.01, α1^-/-^ vs. WT mice, for α2 isoform expression; # p < 0.02, α2^-/-^ vs. WT mice, for α1 isoform expression.

**Figure 4:**
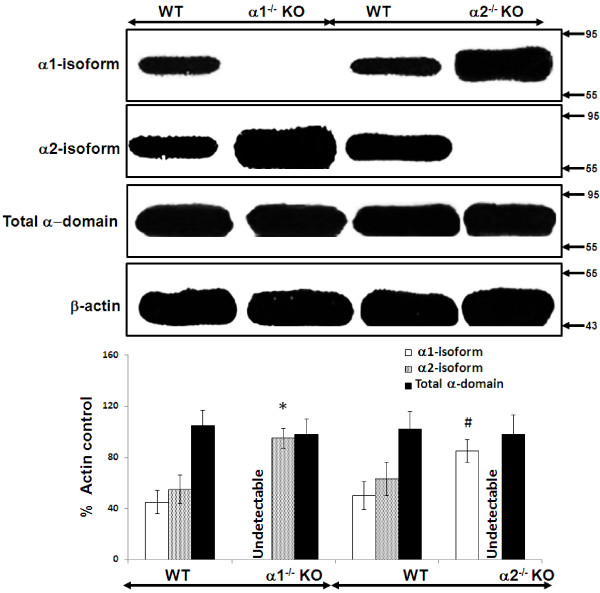
**Relative expression of the catalytic alpha (α) domain of AMPK in proximal tubular cells derived from α1**^**-/- **^**and α2**^**-/- **^**knockout (KO) mice.** The relative expression of the α1 and α2 isoforms of AMPK, as well as of the total α domain (α1 + α2), was evaluated in immunoblots of lysates from confluent mouse proximal tubular (MPT) cells derived from the kidneys of either α1^-/-^ or α2^-/-^ KO mice, or their WT controls. Upper panel: Representative immunoblot. Molecular weight markers (in kDa) are shown on the right of each immunoblot. Lower panel: Densitometric quantitation of three immunoblots using β-actin as a loading control. * p < 0.02, α1^-/-^ vs. WT mice, for α2 isoform expression; # p < 0.02, α2^-/-^ vs. WT mice, for α1 isoform expression.

We used immunoblotting to determine the relative expression of the α1 and α2 isoforms of AMPK, as well as total α domain expression (α1 + α2 isoforms), in lysates obtained from whole kidney cortex (Figure 3) and confluent monolayers of MPT cells (Figure 4), both derived from AMPK KO mice and their WT controls. As expected, we detected no expression of the deleted α1 or α2 isoform in lysates from either whole kidney cortex (Figure 3) or MPT cell cultures (Figure 4) obtained from the α1^-/-^ and α2^-/-^ mice, respectively. Notably, however, expression of the non-deleted α-isoform was markedly increased in kidney cortex and MPT cell cultures from AMPK KO mice when compared to their WT controls (Figures 3 and 4). As a result, total alpha domain expression was comparable in kidney cortex and MPT cell cultures for both types of AMPK KO mouse versus its WT controls (Figures 3 and 4).

### Effect of metabolic stress on activation of the AMPK pathway in MPT cells from α1^-/-^ and α2^-/-^ mice

We next compared the degree to which AMPK is activated by metabolic stress in MPT cells from AMPK KO versus WT mice. Metabolic stress was induced by treatment of cells with antimycin in the presence of 5 mM dextrose. Activation of AMPK was assessed by immunoblotting for phosphorylation of Thr^172^ within the catalytic α domain of AMPK. Upon activation, AMPK phosphorylates a number of downstream targets [1,3-5]. As a further measure of AMPK activation, we evaluated the extent of phosphorylation of ACC at Ser^79^, an event that inhibits ACC activity. We chose ACC, since it is one of the more thoroughly studied downstream targets of AMPK [1].

**Figure 5:**
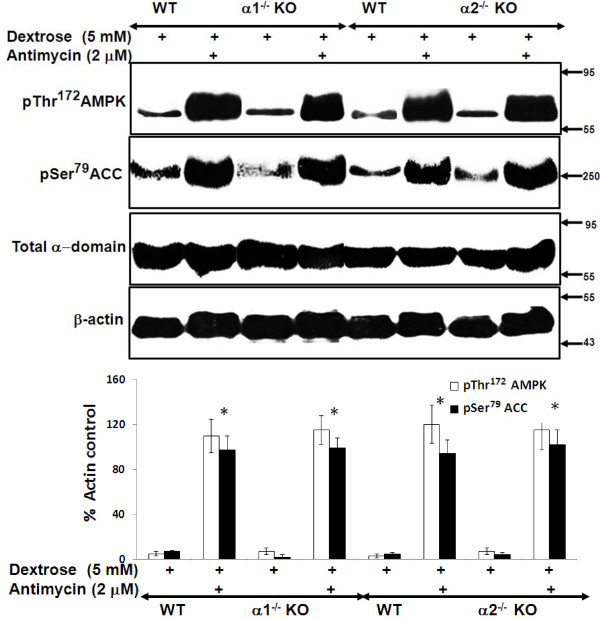
**Effect of metabolic stress on activation of the AMPK pathway in MPT cells from α1**^**-/- **^**and α2**^**-/- **^**KO mice.** MPT cells obtained from α1^-/-^ or α2^-/-^ KO mice, or their WT controls, were subjected to metabolic stress by incubation in medium containing dextrose (5 mM) plus antimycin (2 μM) for 4 hrs. Control cells were incubated in dextrose in the absence of antimycin. Activation of the AMPK pathway was assessed by immunoblotting using antibodies that recognize either the phosphorylated (activated) form of AMPK or the phosphorylated (inhibited) form of acetyl coenzyme A carboxylase (ACC). Expression of total AMPK was also assessed. Upper panel: Representative immunoblot. Molecular weight markers (in kDa) are shown on the right of each immunoblot. Lower panel: Densitometric quantitation of three immunoblots using β-actin as a loading control. * p < 0.001, presence vs. absence of antimycin in MPT cells from α1^-/-^ mice, α2^-/-^KO mice and their WT controls.

Treatment with antimycin increased phosphorylation of both AMPK and ACC to a comparable extent in MPT cells from α1^-/-^ and α2^-/-^ mice versus their WT controls (Figure 5). These data suggest that the identity of the alpha domain isoform does not influence the degree to which AMPK is activated by antimycin-induced metabolic stress, and that each isoform can substitute for the absence of the other.

### Effect of pharmacologic inhibition of AMPK on stress-induced activation of AMPK in MPT cells from AMPK KO and WT mice

**Figure 6:**
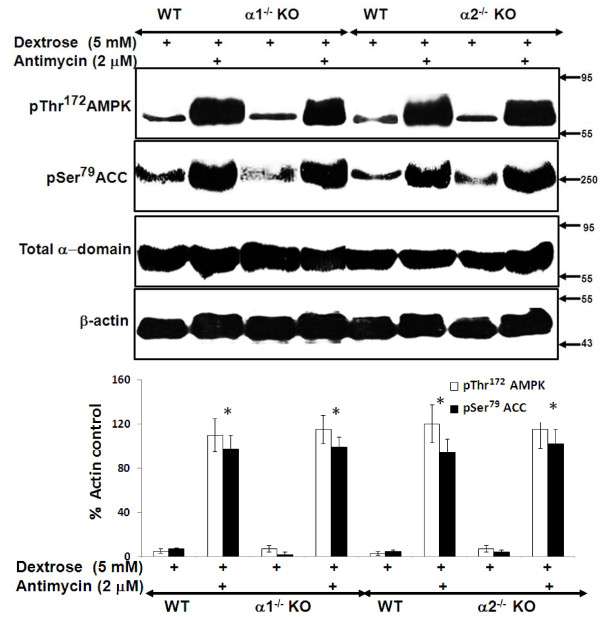
**Effect of pharmacologic inhibition of AMPK using compound C (CC) on the activity of the AMPK pathway in MPT cells from α2**^**-/- **^**KO and WT mice.** MPT cells from α2^-/-^ mice, or their WT control, were subjected to ATP depletion by incubation in medium containing dextrose (5 mM) and antimycin (2 μM), in the presence or absence of CC (20 μM) for 4 hrs. Activation of the AMPK pathway was assessed by immunoblotting, using antibodies that recognize either the phosphorylated (activated) form of AMPK, or the phosphorylated (inhibited) form of ACC. Expression of total alpha domain was also assessed. Upper panel: Representative immunoblot. Molecular weight markers (in kDa) are shown on the right of each immunoblot. Lower panel: Densitometric quantitation of three immunoblots using β-actin as a loading control. * p < 0.01, presence vs. absence of CC, for MPT cells from WT mice treated with antimycin; # p < 0.01, presence vs. absence of CC, for MPT cells from α2^-/-^ mice treated with antimycin.

We examined the effect of compound C (CC), a pharmacologic inhibitor of AMPK, on AMPK activity during metabolic stress in MPT cells from α2^-/-^ and WT mice. Antimycin increased the phosphorylation of both AMPK and its downstream target, ACC, in MPT cells from α2^-/-^ and WT mice (Figure 6). CC comparably reduced the stress-induced increase in phosphorylation of AMPK and ACC in MPT cells from α2^-/-^ versus WT mice (Figure 6). Similar results, in the absence and presence of CC, were obtained in MPT cells from α1^-/-^ versus WT mice (data not shown).

### Effect of pharmacologic inhibition of AMPK on viability of metabolically stressed MPT cells from AMPK KO and WT mice

**Figure 7:**
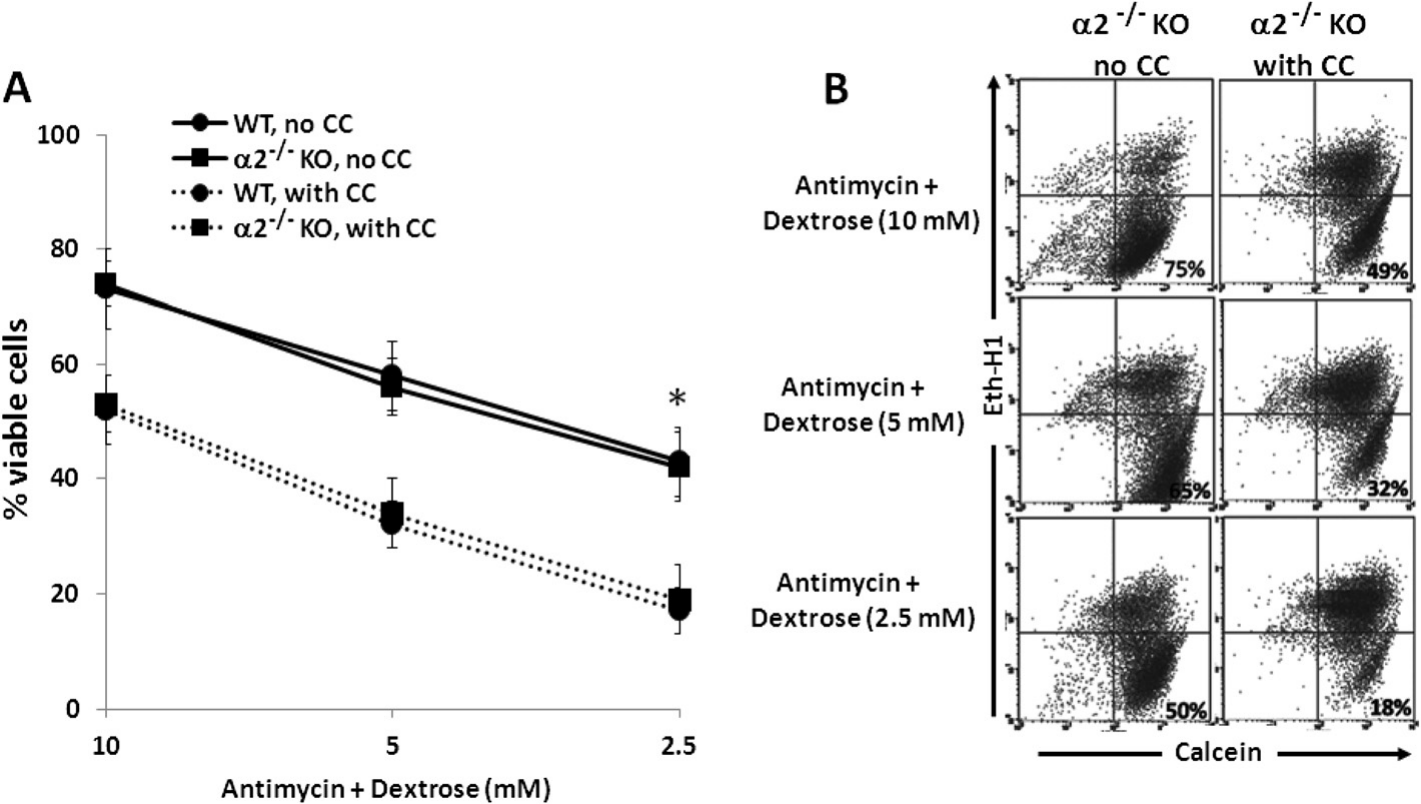
**Effect of pharmacologic inhibition of AMPK on viability of MPT cells from α2**^**-/- **^**KO and WT mice subjected to metabolic stress. A**: MPT cells from α2^-/-^ mice, or their WT control, were subjected to graded ATP depletion by incubation in the presence of antimycin (2 μM) and varying concentrations of dextrose for 16–18 hrs, in either the absence (solid lines) or the presence (dashed lines) of CC (20 μM). The percentage of viable cells remaining was determined by flow cytometry (n = 5). * p < 0.01, absence vs. presence of CC, for MPT cells from either α2^-/-^ mice or their WT control (ANOVA for repeated measures). **B**: Representative flow cytometric analysis of metabolically stressed MPT cells from α2^-/-^ mice incubated in the absence (left panels) or presence (right panels) of CC (20 μM). Cell viability was quantified by staining cells with calcein AM (x-axis) and ethidium homodimer-1 (Eth-H1) (y-axis). For each condition, 10,000 cells were analyzed, and the percentage of viable cells was calculated by dividing the number of calcein-positive and Eth-H1-negative cells by the total number of cells counted.

We next examined the effect of CC on MPT cell viability during metabolic stress. MPT cells from α2^-/-^ mice and their WT controls were subjected to graded ATP depletion (antimycin in the presence of varying concentrations of dextrose) for 16–18 hrs, in the presence of either CC (20 μM) or its vehicle. Control cells were incubated in dextrose without antimycin. In the absence of CC, metabolic stress induced a comparable decrease of viability in MPT cells from α2^-/-^ versus WT mice (Figure 7). Although inhibition of AMPK with CC reduced MPT cell viability at all degrees of metabolic stress, the reduction in viability induced by CC was comparable in MPT cells from α2^-/-^ versus WT mice (Figure 7). Furthermore, regardless of the presence or absence of CC, ATP levels in metabolically stressed MPT cells did not differ for α2^-/-^ versus WT mice (data not shown). These data are consistent with those of immunoblotting (Figures 5 and 6).

We performed similar experiments using MPT cells from α1^-/-^ versus WT mice. In the absence of CC, metabolic stress (antimycin plus 5 mM dextrose) reduced cell viability to a similar extent in MPT cells from α1^-/-^ versus WT mice (64 ± 7% and 59 ± 8% of control, respectively). CC comparably exacerbated the stress-induced loss of cell viability in MPT cells from α1^-/-^ versus WT mice (35 ± 6% and 38 ± 7% of control, respectively). Thus, the effects of CC on the viability of stressed MPT cells was similar for α1^-/-^ and α2^-/-^ mice, as compared to their WT controls.

### Effect of knockdown of the α2 isoform on expression and activation of the AMPK pathway in α1^-/-^ and WT mice

**Figure 8:**
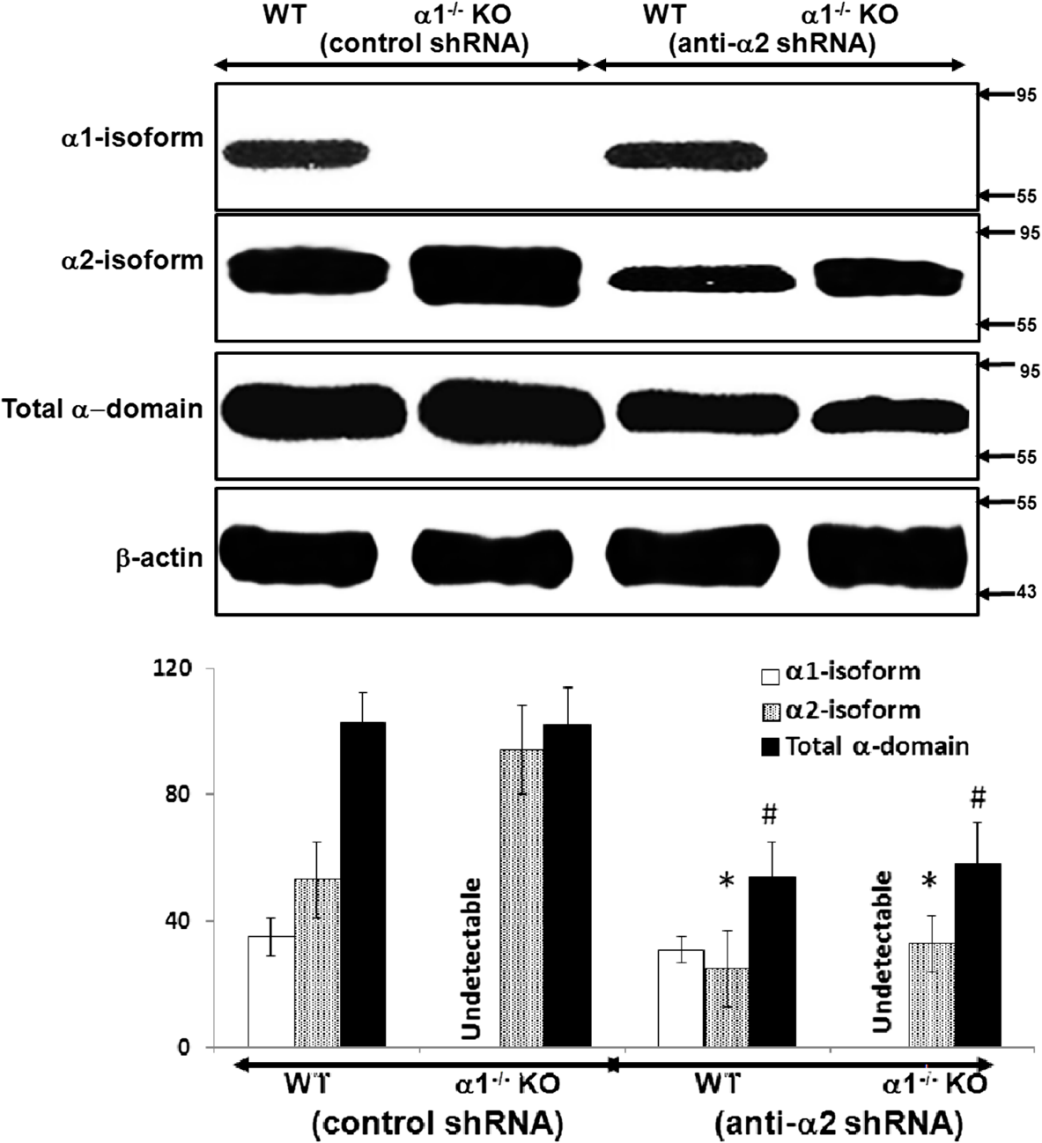
**Effect of knockdown of the α2 isoform on expression of the α1 and α2 isoforms and of total alpha domain in MPT cells from α1**^**-/- **^**and WT mice.** MPT cells from α1^-/-^ mice, or their WT control, were infected with control (scrambled) shRNA or anti-α2 shRNA designed to knock down the expression of the α2 isoform of AMPK. The relative expression of the α1 and α2 isoforms of AMPK, as well as of the total α domain (α1 + α2), was evaluated in immunoblots of lysates from confluent MPT cells derived from the kidneys of α1^-/-^ and α2^-/-^ mice, and their WT controls. Upper panel: Representative immunoblot. Molecular weight markers (in kDa) are shown om the right of each immunoblot. Lower panel: Densitometric quantitation of three immunoblots using β-actin as a loading control. * p < 0.05, anti-α2 shRNA vs. control shRNA, for α2 isoform expression in MPT cells from WT and α1^-/-^ mice; # p < 0.05, anti-α2 shRNA vs. control shRNA, for total alpha domain expression in MPT cells from WT and α1^-/-^ mice.

MPT cells from α1^-/-^ or WT mice were infected with either control (scrambled) shRNA or shRNA designed to knock down expression of the α2 isoform of AMPK. Infection with control shRNA did not alter expression of either the α2 isoform of AMPK or total α-domain AMPK in MPT cells from α1^-/-^ and WT mice (Figure 8). In contrast, infection with anti-α2 shRNA significantly reduced expression of the α2 isoform of AMPK as well as total alpha domain AMPK in MPT cells from both α1^-/-^ and WT mice (Figure 8).

**Figure 9:**
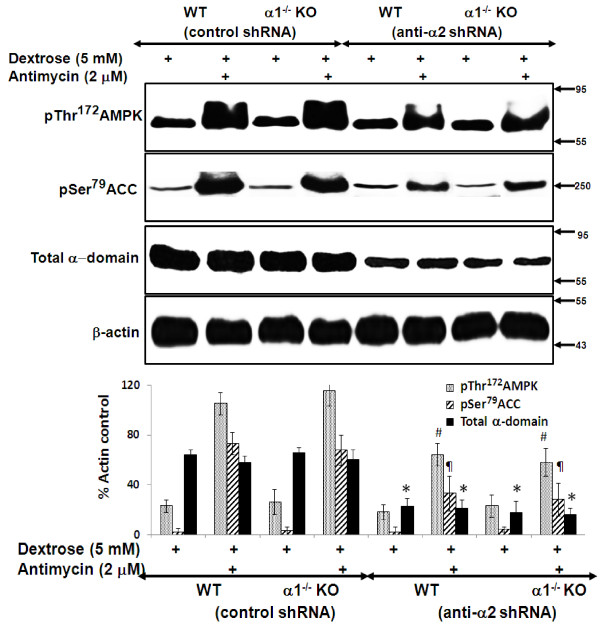
**Effect of knockdown of the α2 isoform of AMPK on the activity of the AMPK pathway in MPT cells from α1**^**-/- **^**and WT mice.** MPT cells from α1^-/-^ mice, or their WT control, were infected with control shRNA or anti-α2 shRNA designed to knock down the expression of the α2 isoform of AMPK. MPT cells were subjected to metabolic stress by incubation in medium containing dextrose (5 mM) plus antimycin (2 μM) for 4 hrs. Control cells were incubated in dextrose in the absence of antimycin. Activation of the AMPK pathway was assessed by immunoblotting using antibodies that recognize either the phosphorylated (activated) form of AMPK or the phosphorylated (inhibited) form of ACC. Expression of total AMPK was also assessed. Upper panel: Representative immunoblot. Molecular weight markers (in kDa) are shown om the right of each immunoblot. Lower panel: Densitometric quantitation of three immunoblots using β-actin as a loading control. * p < 0.01, anti-α2 shRNA vs. control shRNA, for total α domain expression in MPT cells from WT or α1^-/-^ mice; # p < 0.02, anti-α2 shRNA vs. control shRNA, for phospho-AMPK expression in MPT cells from WT or α1^-/-^ mice; p < 0.02, anti-α2 shRNA vs. control shRNA, for phospho-ACC expression in MPT cells from WT or α1^-/-^ mice.

We next determined the effect of knockdown of the α2 isoform of AMPK on metabolic stress-induced activation of the AMPK pathway. MPT cell monolayers from α1^-/-^ and WT mice were infected with either control or anti-α2 shRNA. Following infection, MPT cells were incubated in dextrose (5 mM) in the absence or presence of antimycin. In cells infected with control shRNA, metabolic stress induced marked phosphorylation of both AMPK and ACC (Figure 9). Infection with anti-α2 shRNA led to a marked but comparable reduction in phosphorylation of AMPK and ACC in MPT cells from α1^-/-^ versus WT mice (Figure 9).

### Effect of knockdown of the α2 isoform of AMPK on viability of metabolically stressed MPT cells from α1^-/-^ KO mice

**Figure 10:**
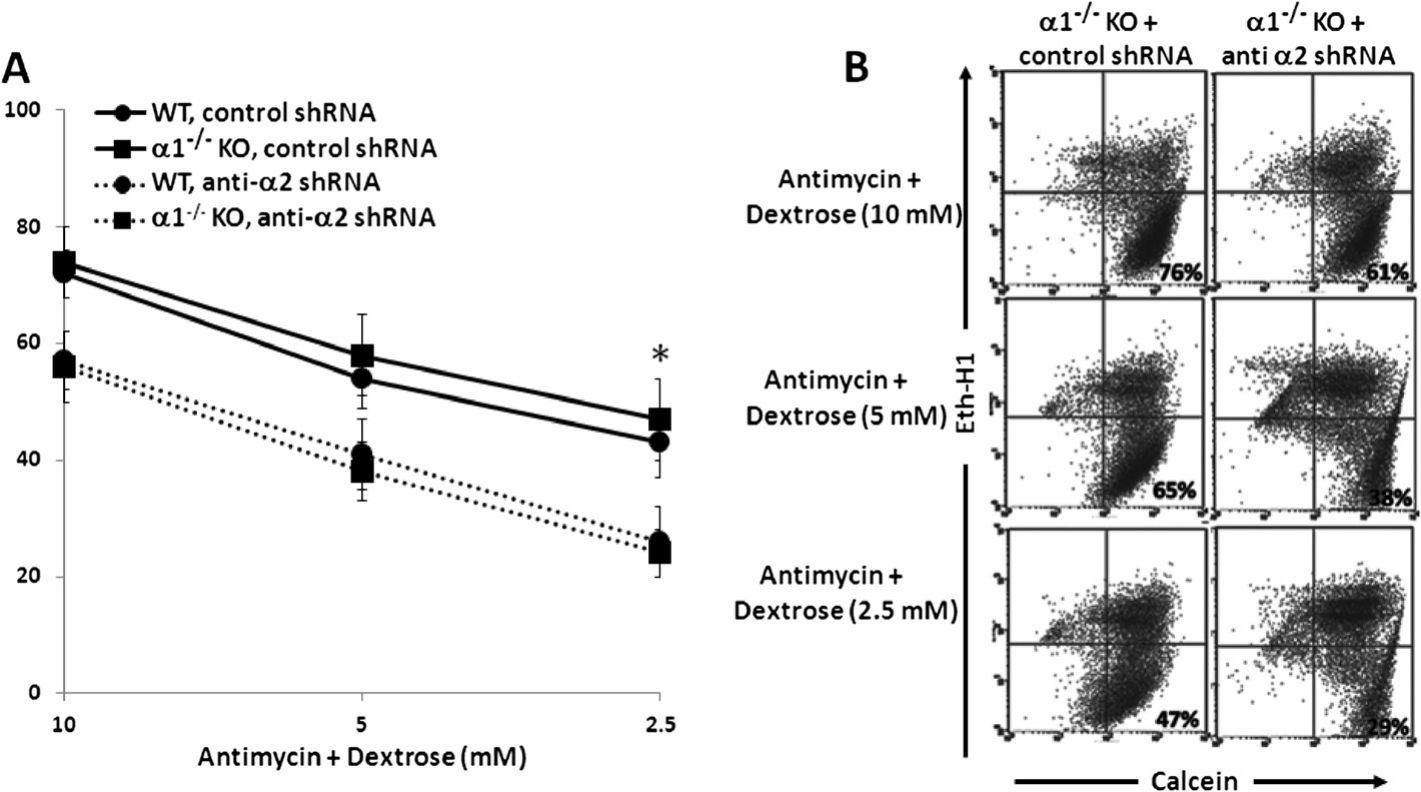
**Effect of knockdown the α2 isoform of AMPK on the viability of MPT cells from α1**^**-/- **^**KO and WT mice subjected to metabolic stress. A**: MPT cells from α1^-/-^ mice, or their WT control, were infected with control shRNA (solid lines) or anti-α2 shRNA designed to knock down the expression of the α2 isoform of AMPK (dashed lines). Cells were then subjected to graded ATP depletion by incubation for 16–18 hrs in the presence of antimycin (2 μM) and varying concentrations of dextrose. The percentage of viable cells remaining was determined by flow cytometry (n = 5). * p < 0.01, anti-α2 shRNA vs. control shRNA, for MPT cells from α1^-/-^ or WT mice (ANOVA for repeated measures). **B**: Representative flow cytometric analysis of metabolically stressed MPT cells from α2^-/-^ mice infected with anti-α2 shRNA (right panels) or control shRNA (left panels). Cell viability was quantified by staining cells with calcein AM (x-axis) and ethidium homodimer-1 (Eth-H1) (y-axis). For each condition, 10,000 cells were analyzed, and the percentage of viable cells was calculated by dividing the number of calcein-positive and Eth-H1-negative cells by the total number of cells counted.

Finally, we examined the effect of knockdown of the α2 isoform of AMPK on the viability of metabolically stressed MPT cells. MPT cells from α1^-/-^ and WT mice were infected with either control shRNA or anti-α2 shRNA, and then incubated in the absence or presence of antimycin plus varying concentrations of dextrose. After 16–18 hrs, the percentage of viable cells was determined by flow cytometry. As with pharmacologic inhibition of AMPK by CC, knockdown of the α2 domain comparably decreased the viability of metabolically stressed MPT cells from α1^-/-^ versus WT mice (Figure 10). Regardless whether cells were infected with control or anti-α2 shRNA, ATP levels did not differ in metabolically stressed MPT cells from α1^-/-^ versus WT mice (data not shown). Taken together, our studies on the effects of AMPK inhibition, accomplished either pharmacologically or molecularly, demonstrate antimycin-induced metabolic stress comparably activates the α1 and α2 isoforms of AMPK, and that either isoform can substitute for the other in ameliorating stress-induced cell death.

## Discussion

Increased activity of AMPK contributes to the pathogenesis of a wide range of diseases, including diabetes mellitus, the metabolic syndrome, neurodegenerative disorders, and cancer [24-30]. However, while an abundance of data indicates that AMPK is activated across multiple cell types during acute ischemic or toxic injury, the contribution by AMPK to cell survival during and following acute injury remains uncertain. Some studies have reported a cytoprotective role for AMPK [31-35], while others have found that AMPK contributes to cell death [12,36-43]. The net effect of AMPK on cell survival likely depends upon multiple variables, including cell lineage, the nature of the toxic stimulus, and the specific pathways responsible for AMPK activation. In the kidney, few data are available, regarding either the state of AMPK activity following ischemic injury, or the role of AMPK in modulating renal tubular cell survival during metabolic stress.

Previously, we have shown that inhibition of AMPK, either pharmacologically (using CC) or via knockdown techniques, increased apoptosis of an immortalized MPT cell line subjected to metabolic stress [18]. These results suggested an anti-apoptotic role for AMPK in metabolically stressed kidney cells. In this study, we tested the hypothesis that primary cultures of MPT cells, derived from AMPK KO mice lacking either the α1 or α2 isoform of the catalytic domain, would be more susceptible to apoptosis induced by metabolic stress than primary MPT cell cultures from their WT controls. To our surprise, stress-induced death was no more severe in MPT cells from KO mice compared MPT cells from the WT controls (Figure 1). Moreover, while treatment of MPT cells with antimycin in the presence of decreasing concentrations of dextrose led to a progressive fall in cell ATP levels (Figure 2), the decrease in cell ATP levels at every degree of metabolic stress was comparable in KO versus WT mice (Figure 2).

Our data suggest that the lack of difference in susceptibility to stress-induced death by MPT cells from AMPK KO versus WT mice is related to a compensatory increase in the expression of the non-deleted alpha isoform that occurs in the kidney cortex (Figure 3) and MPT cell cultures (Figure 4) from α1^-/-^ and α2^-/-^ mice. Thus, expression of the α2 isoform of AMPK is up-regulated in the cortex (Figure 3) and primary MPT cell cultures (Figure 4) from the kidneys of α1^-/-^ mice. Similarly, expression of the α1 isoform is up-regulated in the cortex (Figure 3) and primary MPT cell cultures (Figure 4) from the kidneys of α2^-/-^ mice. As a result, total α-domain expression (α1 + α 2 isoforms) is comparable in AMPK KO versus WT mice.

We next examined the effect of metabolic stress on the phosphorylation, not only of the α-domain of AMPK, but also of ACC, an immediate downstream target of AMPK that has been widely used as a marker of AMPK activity [1]. Interestingly, metabolic stress induced comparable degrees of phosphorylation of AMPK and ACC in MPT cells from α1^-/-^ mice, α2^-/-^ mice and both of their WT controls (Figure 5). These findings suggest that equivalent expression of the total α-domain by KO and WT mice is matched by functional equivalence of AMPK activity.

We suggest that the lack of a difference in susceptibility to antimycin-induced cell death by MPT cells derived from α1^-/-^ and α2^-/-^ mice versus their WT controls is attributable to an adaptive equivalence in the amount and activity of the total alpha isoform of AMPK in MPT cells from the KO and WT mice. We further suggest that that each α isoform can substitute for the other in phosphorylation of downstream targets and in mediating the anti-apoptotic functions of AMPK. This interpretation is supported by studies in which we examined the effects of inhibiting AMPK in primary cultures of MPT cells from the AMPK KO and WT mice. Pharmacological inhibition of AMPK (using CC) of MPT cells from AMPK KO and WT mice, reduced the antimycin-induced phosphorylation of AMPK and ACC (Figure 6), and exacerbated the stress-induced death of MPT cells from the KO and WT mice (Figure 7). However, the extent to which CC inhibited AMPK phosphorylation (Figure 6), or worsened MPT cell death (Figure 7), was not different between MPT cells derived from the KO and WT mice. Similarly, inhibiting AMPK in MPT cells obtained from α1^-/-^ mice and their WT controls by knocking down the α2 isoform using shRNA, decreased antimycin-induced phosphorylation of AMPK and ACC (Figure 9), and exacerbated the amount of death of MPT cells (Figure 10) obtained from both the KO and WT mice to a comparable degree.

Our data show that while genetic deletion of either the α1 or α2 isoform of AMPK does not affect the response of MPT cells to metabolic stress, inhibition of AMPK induced by CC or molecular knockdown markedly increases the susceptibility of MPT cells from WT and KO mice to metabolic stress. It is likely that the compensatory increase in expression of the non-deleted α-isoform occurring in AMPK KO mice is due to increased protein synthesis. We speculate that, in the case of acute inhibition of AMPK, either by CC or molecular knockdown, there is insufficient time for a compensatory increase of protein synthesis of alpha isoforms to occur.

It is important to note that our results do not exclude the possibility that the various isoforms of AMPK may differ in role and function in different tissues [1,30]. To date, very little data exist on the consequences of genetic deletion of one isoform on the expression and activity of the other isoform(s). Our results indicate that any attempt to link a particular phenotype (or lack of phenotype) with the absence of one or other of the isoforms of AMPK should be done with caution, since expression and activity of the remaining isoform may be subject to an adaptive up-regulation.

## Conclusions

Our study has several major conclusions. First, primary cultures of mouse proximal tubular (MPT) cells from both α1^-/-^ and α2^-/-^ mice demonstrate an adaptive increase in expression of the intact α-domain isoform of AMPK, such that total α-domain expression is comparable in KO versus WT mice. Second, the α1 and α2 isoforms of AMPK are equally sensitive to metabolic stress, since exposure to antimycin led to comparable increases of AMPK activity in primary MPT cells from α1^-/-^ and α2^-/-^ mice. Third, the α1 and α2 isoforms of AMPK provide equivalent protection from stress-induced cell death, since primary MPT cells from α1^-/-^ and α2^-/-^ mice versus their WT controls were equally susceptible to cell death from ATP depletion. Moreover, the use of compound C to inhibit the activity of the α1 or the α2 isoform in primary MPT cells derived from α2^-/-^ and α1^-/-^ mice respectively, or the inhibition of the α2 isoform in primary MPT cells from α1^-/-^ mice (via molecular knockdown) both exacerbated loss of cell viability in response to ATP depletion to the same degree. Taken together, these data suggest that the α1- and α2-isoforms of AMPK are not only similarly activated by ATP depletion, but also similarly effective in reducing cell death during metabolic stress. The adaptive up-regulation of the intact isoform of AMPK in KO mice is consistent with an overall critical role for AMPK in ameliorating apoptosis of proximal tubular cells in response to acute reductions of cellular ATP during ischemia.

## Abbreviations

α: Alpha; β: Beta; γ: Gamma; ACC: Acetyl coenzyme A carboxylase; ADP: Adenine diphosphate; AICAR: *N*-(β-D-Ribofuranosyl)-5-aminoimidazole-4-carboxamide; AMPK: AMP-activated protein kinase; ATP: Adenine triphosphate; AMP: Adenine monohosphate; KO: Knockout; MPT: Mouse proximal tubule; shRNA: Short hairpin RNA.

## Competing interests

The authors declare that they have no competing interest.

## Pre-publication history

The pre-publication history for this paper can be accessed here:

http://www.biomedcentral.com/1471-2369/14/251/prepub
